# “It gave me a sense of control in a treatment where I felt I had none”: the use of social stories and preferences to reduce the use of restraint during nasogastric tube feeding in young people with anorexia nervosa

**DOI:** 10.3389/fpsyt.2026.1900582

**Published:** 2026-08-20

**Authors:** Fiona Duffy, Rachel Freak, Imogen Peebles, Julie Cockburn, Kibbe Watson, Chloe Manning, Helen West, Amelia Austin

**Affiliations:** 1 Department of Clinical Psychology, University of Edinburgh, Edinburgh, United Kingdom; 2 NHS Lothian, Edinburgh, United Kingdom; 3 Florence Nightingale Faculty of Nursing Midwifery and Palliative Care, King’s College London, London, United Kingdom; 4 Department of Psychology, Glasgow Caledonian University, Glasgow, United Kingdom

**Keywords:** anorexia, autism, eating disorder, nasogastric tube feeding, quality improvement, service evaluation

## Abstract

**Objective:**

Nasogastric tube (NGT) feeding can be used in inpatient care, and in certain incidences is necessary under physical restraint. While at times essential, it is highly distressing and a high proportion of young people who receive NGT feeding under restraint may be Autistic. This service evaluation explored patient and staff perspectives of autism-affirming resources, including social stories and an individual preference sheet, embedded in a larger quality improvement (QI) project to reduce NGT feeding under restraint.

**Methods:**

Nine young people who had received NGT feeding and eleven inpatient staff provided Likert-scale ratings and/or descriptive feedback on whether the resource was helpful and reduced distress. QI data on the frequency of NGT feeding under restraint was analysed using run charts.

**Results:**

Five out of nine young people reported the social stories were helpful, or would have been when they first received NGT feeding. Five out of six clinicians who had used them stated they had reduced distress. All staff and young people who had used the NGT preference sheet to support collaborative care planning (including strategies to reduce sensory overwhelm) found it helpful. These findings were embedded in a larger QI project which saw a reduction in NGT feeding under restraint from a median of 20 to 1.5 a week over one year.

**Discussion:**

The use of social stories and a preference sheet to support collaborative care planning for NGT feeding appears to be acceptable to young people and inpatient staff. These resources could form part of a range of autism-affirming adaptations to treatment with the aim of improving treatment experience and reducing restrictive practice.

## Introduction

Anorexia Nervosa (AN) is an eating disorder (ED) characterised by a significantly low body weight or rapid weight loss, disturbed weight or shape perceptions, and an intense fear of gaining weight (1, 2). It is associated with severe physical impacts including cardiovascular, renal, endocrine, and gastrointestinal complications (3), as well as increased likelihood of death (4). A small proportion of young people may be unable to be treated safely in the community, and an inpatient admission is required. For those who experience ED cognitions so compelling that they still cannot eat with intensive support, and medical stability is precarious, nasogastric tube (NGT) feeding may be recommended (5). NGT feeding refers to the passage of a fine tube via the nasal passage and into the stomach to deliver nutrition (6).

In some incidences, where fear of weight gain and/or food is so great, and when a young person’s physical health is significantly comprised, NGT feeding without consent and under physical restraint may be required to deliver nutrition and ensure the individual’s survival (7). The practice of NGT feeding under restraint is not uncommon: a comprehensive audit of NHS England psychiatric inpatient units estimated 622 patients received NGT feeding across a one-year period, with more than three quarters of these being children and adolescents (8). While NGT feeding is sometimes necessary to ensure the survival of a young person, it can also be a distressing experience for everyone involved (9).

Brinchmann and colleagues (10) reported that nurses delivering NGT feeding under restraint perceived it to be part of life saving care for AN, although some participants reported that they witnessed the use of this practice at times in ways that felt unnecessary and even unethical. Parents and caregivers of young people receiving NGT feeding under physical restraint reported conflicting thoughts and emotions, including understanding the need of the procedure, experiencing it as traumatic, and feeling empathy for staff (11). While little research is available on perspectives of children and young people themselves, Niederman and colleagues (12) reported on the experiences of young patients across two specialist UK ED units who received NGT, although not necessarily with restraint. While some participants reported that NGT feeding had been regrettable at the time but necessary in retrospect, others continued to describe it as unhelpful (12).

In a case series of 143 patients receiving NGT feeding under restraint in England’s psychiatric inpatient units, 33% were identified as Autistic (8). Similarly, a survey specific to paediatric wards in England found that 43% of the children and young people receiving NGT under restraint had an autism diagnosis (13). Another service in Melbourne, Australia found that 65% of young people restrained for NGT feeding were diagnosed or suspected Autistic (14). Given this initial evidence suggesting high representation of Autistic young people receiving NGT feeding under restraint, the well-established raised prevalence of autism and higher autistic traits in patients with AN (15), and the increased risk of inpatient admission for an ED those with higher autistic traits (16), any attempts to improve NGT feeding practices should carefully consider the needs of Autistic young people.

Autism-affirming healthcare frameworks, such as SPACE (Sensory needs, Predictability, Acceptance, Communication, and Empathy) advocate for the adaptation of care to meet the needs of Autistic patients (17). One approach consistent with this framework, particularly facilitating effective communication and enhancing a sense of predictability, is the use of social stories. Social stories present a short narrative using text and illustrations to break down an upcoming event into discrete steps (18). While they were originally developed by Gray and Garand (19) for use in educational contexts, they have more recently been used to improve the experiences of Autistic young people during healthcare interventions (e.g., 20). The goal of social stories within healthcare settings is to reduce anxiety, prepare for sensory input, and support coping (21). They have previously been used to support Autistic young people through potentially distressing medical procedures, like vaccination (22). More recently, social stories have been proposed as a tool to offer clarity and reduce anxiety in Autistic people with EDs (23). Thus, social stories may support Autistic young people with AN to prepare for sensory input and reduce anxiety through an NGT feeding.

Another autism-affirming approach is the use of collaborative and individualised care planning. Specific to NGT feeding, there have been recommendations to jointly care plan strategies to manage distress before, during and after (24). This includes minimising environmental stressors for neurodivergent individuals with EDs (25). Collaboratively planning NGT feeding around individual preferences may facilitate adaptations that align with SPACE (17), such as addressing sensory needs and supporting the emotional wellbeing of Autistic individuals.

It is also possible that efforts to provide more autism-affirming care, such as social stories or collaborative care plans, may benefit all patients with AN. The concept of universal design suggests that what makes something more inclusive for one group, often makes things more accessible for most people (26). Applied to the current context, it seems reasonable to offer all individuals undergoing NGT feeding, regardless of neurotype, access to potentially helpful resources. However, the perspectives of young people and clinicians in relation to such resources has not yet been explored.

The Melville inpatient unit (IPU) is a 12-bed adolescent generic mental health inpatient setting which supports young people aged 12-17 years across the South East of Scotland who are experiencing acute mental illness, including psychosis and eating disorders. The Melville unit is committed to reducing restrictive practice, aligned with Mental Welfare Commission for Scotland (27) recommendations that, where restraint is considered necessary, it should be the minimum required to deal with the agreed risk and applied for the minimum possible time. Across 2025, a quality improvement initiative (QI) with a range of components was conducted in the unit, with the broad aim of reducing the number of NGT feeds using restraint. One of these initiatives was the development of a NGT resource pack in formats that were accessible for a range of communication needs. The pack included social stories and a preference sheet to support personalised care planning, with the aim of both reducing distress associated with NGT feeding, and by proxy, restraint during this process. The objective of the current paper is to:

1. Explore perceptions of the NGT resource pack from the perspectives of young people with AN and inpatient unit staff.
2. Embed these findings within a larger QI project with the aim of reducing NGT feeding under restraint.

## Methods

Like many quality improvement efforts, this project followed a pragmatic epistemology (28), prioritizing approaches that help to solve real world problems. The project included both quantitative elements and descriptive feedback (Likert scale responses and open text). The work conducted was classified as service evaluation, therefore ethical approval was not required. Written consent was obtained from all patients whose anonymized quotes were used in this paper.

### Setting

In 2025, the Melville IPU had 42 admissions, of which 54% (n=23) had a primary diagnosis of AN with a mean Eating Disorder Examination Questionnaire (29) global score of 3.61 (n=16, SD=1.82) on admission. The Melville IPU is in the process of implementing the Pathway for Eating Disorder and Autism developed by Clinical Experience (PEACE: 30) supporting autism affirming approaches to ED care and treatment. The IPU uses the 10-item Autism Spectrum Quotient (AQ-10: 31) to screen for autistic traits on admission, as recommended by the PEACE pathway implementation tool (32). In 2025, 26% (n=6) of young people admitted to the unit with AN had a pre-existing diagnosis of autism and an additional 12% (n=3) were identified as having high autistic traits on the AQ-10.

### NGT feeding resource

Following feedback from young people about the need for collaborative care planning and standardisation of the preparation for NGT feeding, two assistant psychologists (IP, RF) developed an NGT resource pack. They elicited feedback from young people and staff on the potential contents of the resource pack and were informed by autism-affirming practice, including consideration of different communication needs. It was agreed that the resource pack should include 1) a nurse checklist covering NGT feeding preparation with a young person (e.g., what to discuss with the young person, providing opportunities for them to view room and equipment), 2) a young person’s information sheet on commonly asked questions around NGT feeding, 3) social stories on NGT placement and the feeding process, 4) a preference sheet to support the collaborative development of an individualised care plan for before, during and after NGT feeding, 5) a list of common distress tolerance skills. The resource pack can be found in **Supplementary File 1**. The pack was launched in December 2025.

### Participants

Nine young people in the Melville IPU who had received NGT feeding on at least one occasion, either on Melville IPU or another unit (including a paediatric admission) were presented with the NGT resource pack and asked for feedback. Young people were 78% female (n=7) aged 12-17 years old (M=15, SD=2.12). All had a diagnosis of AN and 67% had an autism diagnosis (n=4) or high autistic traits on the AQ-10 (n=2). Eleven multi-disciplinary staff participated in the evaluation, including nursing, dietetics and medical staff. Seventy two percent of staff were female and were aged 23-40 years (M=27.55, SD=4.63).

### Descriptive feedback on NGT educational resource

Young people and staff were asked a series of questions on the social stories and the NGT preference sheet. These included: A) if they had used the resource, B) whether they thought it helped them (or their patients) feel more prepared for NGT feeding, C) whether they thought it reduced any distress around NGT feeding, and D) if they would recommend it to another person in a similar situation. Young people and staff were invited respond to each prompt using a 4-point Likert scale response (from ‘not at all’ to ‘a lot’) and/or to provide open ended feedback. While all staff responded using the Likert scale, most young people preferred to answer using open feedback (verbal comments) with support from an assistant psychologist.

### QI data

The introduction of the NGT resource pack was part of a larger Melville QI project with the aim of reducing the number of NGT feedings under restraint by 20% in 6 months. Improvement and learning efforts across 2025 included a professional development session on reducing restrictive practice (February), launch of a standard operating procedure for ED care (May); a draft NGT standard operating procedure (November); and the introduction of the NGT resource pack (December). This was in addition to staff training across 2025 in Adaptive Mentalization Based Integrative Treatment (AMBIT: 33) which is a team-based approach used to promote reflective practice.

To track the impact of these changes, the frequency and percentage of NGT feeding under restraint was collected by an assistant psychologist (RF) between January and December 2025. The level of support/restraint required during NGT feeding was routinely documented. Records of “support” or “handholding” were not classified as restraint for the purpose of this QI project, however, if a young person required active physical support from nursing staff to enter the treatment room, even if no restraint was required while receiving NGT feeding, this was also operationalised as restraint.

Data were analysed using run charts, which are an appropriate way to examine if the changes made within a healthcare system over time lead to improvements (34). Data points for both the frequency and the percentage of NGT feeding were generated weekly. A ‘shift’ was defined as six or more consecutive data points above or below the median. A baseline median value of NGT feeding with restraint was calculated during the first four weeks of the year, and when a shift occurred, a new median line was plotted. A shift in the data is an indicator on non-random (special cause) variation rather than the random variation that might be expected in a stable system (35). Data is missing from four weeks (one in May and three in July) due to staff absence. These four datapoints were treated as missing observations and not imputed into the run chart.

## Results

### Descriptive feedback on NGT resource pack

#### Social stories

**Young people**. Of the nine young people who provided feedback, four reported that the social stories were provided before receiving NGT feeding. Another four participants had received NGT feeding prior to the launch of the resource pack and were therefore viewing the social story resource for the first time at the point of feedback. One patient wasn’t sure if they had seen it previously or not.

Of the four who were provided with the social stories prior to NGT feeding, two (50%) felt it to be helpful, with one of these young people reporting that the resource “*included details that I hadn’t thought of or realised*.” One young person (25%) reported that they already knew what to expect from previous NGT feeding, and one (25%) said that it was not helpful given that what they had really wanted was to see the treatment room and equipment prior to the procedure.

Of the five young people who had not been provided the social stories during their NGT feeding, or could not recall if they had seen it, three (60%) reported that they would have appreciated the resource the first time they received NGT feeding, with one commenting, “*I had a few before. It would have been helpful the first time.*” Two young people (40%) felt that they would not have benefited from it.

When all nine young people were asked if they would recommend the social story resource to another young person in a similar situation, six (67%) endorsed the approach while three (33%) declined to respond either way. One young person commented: “*if it was someone new, and they were told they were going to get a tube passed it sounds scary.* [The social stories] *provide reassurance and understanding that it’s not supposed to be something horrible*.”

**Staff**. Of the eleven staff members surveyed, six had used the social stories in their previous practice. Of these, two staff members (33%) reported that the resource helped the young person feel a lot more prepared, three (50%) reported that it helped quite a bit, and one (17%) reported that it helped a little. When these six staff members were asked about the reduction of distress in patients, four (67%) reported that social stories reduced the distress of the young person a little bit, one (17%) said a lot, while one (17%) reported not seeing a difference. One of these staff members commented that “*patients have appeared more relaxed during their first treatments as they understand the proces*s” although another noted that they could sometimes “*make the patients more apprehensive as they knew explicitly what was about to happen like the potential of pain from NG tube insertion.*” When staff were asked if they would recommend the social stories to another clinician in a similar situation, all six endorsed the resource, from quite a bit (n=2, 33%) to a lot (n=4, 67%).

#### NGT preference sheet

**Young people**. Of the nine young people who provided feedback, five reported that they had worked through the NGT preference sheet with a nurse to jointly develop a personalised care plan for before, during and after NGT feeding. Three reported that they were not provided with the resource given that it was launched after they had received NGT feeding, and thus were reviewing the preference sheet for the first time at the point of feedback. One patient did not respond to the prompt. Of the five who were provided with the NGT preference sheet, all five (100%) found it to be at least a little helpful, with one young person reflecting that “*it gave me a sense of control in a treatment where I felt like I had none*.”

Of the three young people who were not previously provided with the NGT preference sheet, two (67%) thought it would have been helpful, while one (33%) reported that it was not necessary for them.

When all nine young people were asked if they would recommend the preference sheet to another young person in a similar situation, five (56%) endorsed the approach while four (44%) declined to respond either way. One young person commented on the individualised care plans that were developed following the preference sheet: “*it can reduce distress and anxiety. If someone has a favourite song they want to listen to it can calm them and distract them*.” Another young person further recommended that they thought the preference sheet might have been most helpful on admission, just in case NGT feeding was needed.

**Staff**. Of the eleven staff members surveyed, ten had used the NGT preference sheet to support care planning in their previous practice. Of these, four staff members (40%) reported that the resource helped the young person feel a lot more prepared, four (40%) reported that it helped quite a bit, and two (20%) reported that it helped a little. When these ten staff members were asked about the reduction of distress in patients, five (50%) reported that the preference sheet and associated care plan reduced the distress of the young person a little bit, three reported quite a bit (30%), one said a lot (10%), and one said not at all (10%). One staff member commented that the preference sheet “*allows nurses to collaborate in a way that fosters person centered care”* and were *“able to tailor treatment to patient preferences”* but reinforced that the care plans need to be realistic otherwise it can lead to frustration for young people when they are unable to be implemented. When staff were asked if they would recommend the NGT preference sheet to other clinicians, nine endorsed the resource, from quite a bit (n=2, 20%) to a lot (n=7, 70%), with one non-response.

### Number of NGT feeds under restraint

**Figure 1:**
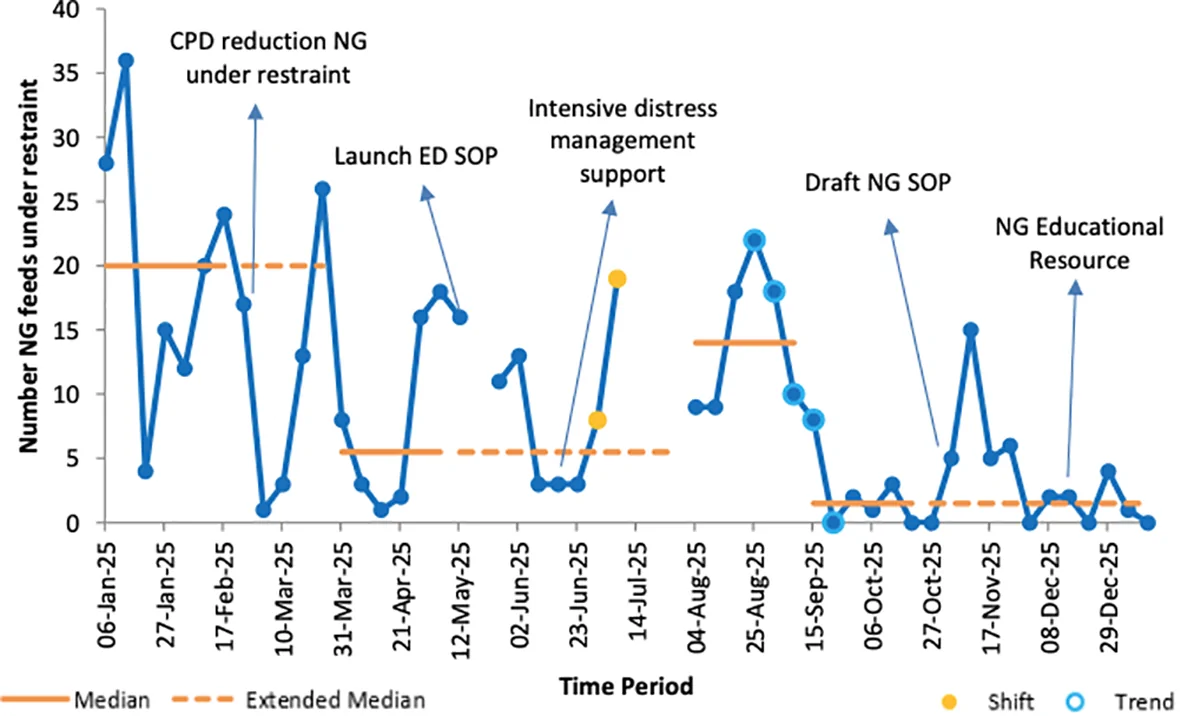
Run chart depicting the number of nasogastric feeds under restraint across one calendar year.

Across the year, the median number of NGT feeds with restraint dropped from 20 per week in January to 1.5 per week from September to December (see **Figure 1**). The shifts observed across the run chart indicate non-random (special cause) variation rather than being attributed to random chance. The median percentage of NGT feeds under restraint (out of all NGT feeds, including those with no restraint required) dropped from 68.3% in January to 10.2% in September through to the end of the year.

## Discussion

Overall, the introduction of an NGT resource pack inclusive of social stories and a preference sheet to support individualised care planning before, during and after NGT feeding, was generally well received by young people and inpatient unit staff. Most young people reported that they found social stories on NGT insertion and feeding helpful or would have appreciated it the first time they received NGT feeding. These views were mirrored by inpatient staff, the majority of whom felt that the social stories supported young people to feel more prepared for the procedure and reduced distress. These findings replicate the introduction of social stories to prepare young people for other medical procedures (20, 22). However, personal preference was noted, with one young person stating they would have preferred to have viewed the treatment room and handled a feeding tube instead of using social stories, and a staff member reporting that for some young people, explicit information, especially information related to potential pain, increased apprehension of the process. This reinforces the need for social stories to be used as part of a suite of resources, which can be selected based on a young person’s preferences and needs.

There was endorsement from several young people of the use of a preference sheet to support individualised care plans before, during and after NGT feeding. Staff equally supported the use of this resource but provided the caveat that the care plans needed to be realistic to prevent frustration for young people if they cannot be implemented. One young person recommended that the preference sheet might have been most helpful on admission. This recommendation is mirrored by Fuller and colleagues (24) who highlighted that many discussions around NGT feeding occur at points of crisis, when a young person is significantly struggling with oral intake and there are medical risks. They suggest that good practice would be advanced care planning at the start of admission, so young people are clear about what NGT feeding involves and when restrictive practice may be needed. Indeed, Fuller et al. (24) recommends that this is extended to involve patients, their families, and the MDT to discuss what helps a patient manage their required nutrition to *avoid* NGT under restraint. However, further evaluation of NGT advanced care planning is required as there is potential for iatrogenic harm, e.g., young people who would not have received NGT feeding being introduced to this as a potential intervention and therefore viewing this as a natural course of admission, validation of the severity of their illness and/or NGT feeding being perceived as a threat.

While initial feedback on these resources has been generally positive, these tools are not intended to be used in isolation, but as part of a range of autism-affirming adaptions, such as those advocated for by the PEACE pathway (30). Implementation of the PEACE pathway includes consideration of autistic communication, cognitive and sensory needs across different aspects of ED treatment and care (32). Furthermore, accommodating patients’ sensory needs in general mental health units has been shown to decrease the number of restraint episodes (36). Autism-affirming adaptions in inpatient unit care that are designed to facilitate oral intake of nutrition, and strategies to reduce sensory overwhelm such as co-designed sensory rooms (25, 36) need to be implemented *in addition* to NGT feeding resources, to prevent the need for NGT feeding and restrictive practice where possible.

This is the first service evaluation to explore perceptions of NGT feeding resources for young people with AN in an inpatient setting. Its strength is that the resource was developed in partnership with young people and focuses on autism-affirming and collaborative person-centered care. However, there are also limitations to this service evaluation. While the use of Likert scales was encouraged, young people’s preference was to give verbal feedback meaning there are gaps in the data presented. Four out of the nine young people had not received social stories as part of their care as they had experienced NGT feeding prior to the resource packs’ launch, meaning they were able to give feedback on the resource, but were unable to comment specifically on its impact during NGT feeding. Moreover, while most staff had implemented the preference sheet in their clinical work, several had not yet implemented the social story resource, limited the amount of feedback we were able to obtain on the latter. Furthermore, the QI data presenting a reduction in NGT feeding under restraint cannot be attributed solely to the resource pack, as most of the changes had been noted prior to its introduction. However, QI methodology supports iterative tests of change to refine broader quality improvement in healthcare and reducing restrictive practice in inpatient units needs to be an ongoing and intentional process. National data collection of NGT feeding under restraint across inpatient units that is conduced sensitively could facilitate shared learning and a collaborative and supportive approach to reducing restrictive practice.

Overall, the use of social stories and a preference sheet to support collaborative care planning for NGT feeding appears to be acceptable to both young people with AN and inpatient staff. These resources could form part of a range of autism-affirming adaptations to ED treatment and care in inpatient units with the aim of improving quality of care, treatment experience and the potential to reduce restrictive practice.

## Data Availability

The data supporting the conclusions of this study is not publicly available due to ethical restrictions. Requests to access these datasets should be directed to fiona.duffy@ed.ac.uk.
