# Supplementary material for: “It gave me a sense of control in a treatment where I felt I had none”: the use of social stories and preferences to reduce the use of restraint during nasogastric tube feeding in young people with anorexia nervosa
Source: Front Psychiatry. 2026 Aug 20;17:1900582. doi: 10.3389/fpsyt.2026.1900582 (PMC13540073; doi:10.3389/fpsyt.2026.1900582)
Supplement: Supplementary file 1 [file SupplementaryFile1.docx]

Nasogastric feeding resource

Contents

[Checklist for preparing young people for Naso-Gastric feeding	2](#_Toc234338592)

[NG tube feeding guide for young people	3](#_Toc234338593)

[Questions you might have	3](#_Toc234338594)

[Social Story 1 What is a Meal Plan?	5](#_Toc234338595)

[Social Story 2 Having a Naso-gastric (NG) tube fitted	7](#_Toc234338596)

[Social Story 3. What happens when I have a NG Feed?	16](#_Toc234338597)

[Support during Naso-Gastric feeding	21](#_Toc234338598)

[Preferences before, during and after NG feed	21](#_Toc234338599)

[Before the NG feed	21](#_Toc234338600)

[Going to the NG feed	21](#_Toc234338601)

[During the NG feed	22](#_Toc234338602)

[After the NG feed	23](#_Toc234338603)

[Strategies that can help me throughout the process	25](#_Toc234338604)

# Checklist for preparing young people for Naso-Gastric feeding

This checklist can be used to help prepare young people for Naso-Gastric (NG) feeding. It might be helpful to go through with young people who are going to receive NG feeding to ensure they understand why they need NG feeding and to prepare them for the procedure.

***Before* speaking to the young person**

- ☐The young person has been offered all alternatives forms of nutrition (Meal plan and supplement) before discussing NG tube feeding.
- ☐It has been confirmed the agreed plan within the MDT is to offer NG feeding.

**Explain the need for NG feed**

- ☐Explain why the young person is being offered NG tube feeding.
- ☐Take time to ensure that the young person is clear about the physical risks to their health and why they require feeding via a NG tube.
- ☐ Remind the young person that NG feeding is a last resort and short-term.
- ☐ Explain to the young person in terms they understand that the MDT will have reached the decision to begin NG feeding (e.g., following a comprehensive risk assessment of physical, nutritional and psychological factors.)

**Explain what an NG tube feeding is**

- ☐Explain what an NG tube is and how it is used for feeding. For example, where it sits in the body, how feed is delivered to the stomach.
- ☐Explain that the NG tube will be fitted, include details about when and by whom.
- ☐Clarify that the feed will be administered at the stage of the NG tube being fitted or at a later point in the day depending on care plan.
- ☐Explain that they always be offered food, then supplement and then the NG feed as a last resort.
- ☐ Highlight that they will always be encouraged to eat orally.

**Demonstrating the NG tube**

- ☐Explain how a NG tube will fitted and be measured.
- ☐Potentially show a diagram of how NG is placed.
- ☐ Offer to take them to the treatment room and show them relevant materials including show them the NG tube.
- ☐ Offer nasal spray or other alternations to ensure they are comfortable.

**Provide a description of the NG feed procedure**

- ☐Explain when they will be offered NG feed. E.g., post meals or at a set time.
- ☐If possible, walk them through the procedure step by step. Explain fitting the tube, the aspirate, supplement, flush following the feed.
- ☐If appropriate, use the social stories about NG tube fitting and feeding to explain the process. Alternatively, there are a page of FAQs which could be offered.
- ☐Check that they understand how the feed will be administered. Remind them that it can be discussed again at another time if they become distressed.

**Go through their preferences with them**

- ☐Ask about their preferences about the NG tube placement (e.g., which nostril, timing of fitting, leaving the NG tube in)
- ☐Ask about their support preferences before, during and after their NG feed.
- ☐Make suggestions from the preferences handouts.
- ☐Use the support handouts in the resource which include suggestions and guiding questions if helpful.
- ☐Try to accommodate preferences as far as possible. There may be instances where this is not possible. If appropriate, explain to them why this is the case.

**Try to avoid**

- ☐Conversations around volume or the amount of supplement.
- ☐The use of restraint.

# NG tube feeding guide for young people

You have been given this resource as you might need NG feeding for the treatment of your eating disorder. We understand that it can be overwhelming part of your treatment. Hopefully this guide will help you to understand the procedure. You may go through this with staff.

## Questions you might have

### What is nasogastric feeding?

- Nasogastric feeding is sometimes called NG feeding. NG feeding is passing liquid nutrition by a syringe through a tube placed in from your nostril into your stomach.

### Why might I need to have NG feeding?

- There are lots of reasons why someone might need NG feeding. It might be that at the moment you are not able to manage enough nutrition by eating and drinking. You may need to be NG fed as you have not been able to get the nutrition that your body needs. This may mean that there is a risk to your physical health. The care team has a duty for your care, including for your physical health.

### How is the NG tube fitted?

- The NG tube is passed up into your nostril and down through the back of your throat. The tube goes down into your stomach. Staff will fit the tube for you. They may use gel to help the NG tube be inserted more easily.

### What does an NG tube feel like?

- The NG tube should not be uncomfortable. You will be aware of having the tube in your nose and throat. It will be positioned safely by staff. If the NG tube is uncomfortable, staff can try to ease this with nasal spray or by shifting the tube into a more comfortable position. Staff will draw some liquid up through the tube from your stomach. They will test the sample to ensure it is in the correct position in the stomach.

### How does it deliver nutrition?

- Liquid nutrition is taken up by a syringe and pushed through the tube into your stomach.

### Will the NG tube be left in place?

- You will have some choice as to whether the tube is left in place or taken out. This may be decided by staff depending on your circumstances.

### Can I eat and drink with the NG tube in place?

- Yes, you can eat and drink with a NG tube in place.

### How will this happen?

- You will have an NG tube fitted by a staff. When you need an NG feed, nutritional supplement will be passed through the NG tube into your stomach.

The image below shows a cross section of a body with a NG tube.

The image below is of a person with NG tube in place. The syringe attached is delivering feed.

Photo of an NG tube

# Social Story 1 What is a Meal Plan?

| I am in the Melville Inpatient Unit to treat an eating disorder. |  |
| --- | --- |
| I have not been eating enough of the foods my body needs, because of an eating disorder. |  |
| Food is fuel for our bodies, it gives our body and brain energy to grow, to move and to think. |  |
| To treat an eating disorder, I will have a meal plan. The first part of the plan is being offered food to eat. |  |
| I will try to eat the food. When I do not eat it, I will be offered a liquid food replacement, called a nutritional supplement. |  |
| When I do not drink the nutritional supplement, the staff will look at my care plan to let me know what happens next. |  |

# Social Story 2 Having a Naso-gastric (NG) tube fitted

| I have tried to eat the food and the nutritional supplement. Staff have looked at my care plan. |  |
| --- | --- |
| I am getting an NG tube fitted. This is because I need liquid food to give my body and brain fuel. |  |
| I go to the treatment room to have the NG tube fitted. |  |
| Sometimes, I have medication before I go to the treatment room. This will be given between 30 minutes to an hour before. |  |
| A NG tube is a narrow tube that goes into my nose, down my food pipe, called my oesophagus, to my stomach. |    |
| Liquid food goes into the NG tube and down into my stomach, this gives me fuel for my body and brain. |  |
| The staff will usually set up the NG tube, liquid food and supplies in the treatment room. |  |
| I can choose which nostril I want the NG tube to go in to. |  |
| Sometimes, the staff will put a piece of tape over the NG tube and on my cheek to keep it in place. |  |
| Usually, the staff will hold the NG tube to my nose. |  |
| Usually, the staff will then wrap the NG tube around my ear and measure it down to my belly. |  |
| This helps the staff know how long the NG tube needs to be. |  |
| The staff will put some clear gel on the end of the NG tube. |  |
| This usually helps the NG tube slide into my nose easily. |  |
| I am now ready for the NG tube to be put in. I can talk through my preferences with staff and fill out an NG preferences sheet. |  |
| I will try to keep still while the NG tube is put in. |  |
| The staff will slide the NG tube into my nose. |  |
| I will probably feel the tube in the back of my throat. |  |
| Usually, the staff will ask me to take a sip of a drink, this will help the tube go down my food pipe to my stomach. |  |
|  |  |
| The staff will attach a small syringe to the end of the tube. |  |
| This helps the staff check that the end of the NG tube is in my stomach. |  |
| The syringe stays attached to the end of the tube. |  |
| The staff will pull on the syringe to collect liquid from my stomach. |  |
| When the NG tube has reached my stomach, staff will tape the tube to my cheek. |  |
| They will put a cap at the end of the tube to keep it closed and seal it with tape. | Picture# |
| This helps the tube stay in the right place. |    |
| I am all done. |  |
| The NG tube has been fitted. |  |
| I can ask the staff questions I might have about the NG tube and the food going into my stomach and they will try to answer them. |  |

# Social Story 3. What happens when I have a NG Feed?

| I will usually go with a member of staff to the treatment room. |  |
| --- | --- |
| Usually, I will sit on the edge of the bed in the treatment room. |  |
| The staff will offer me support how I choose. I can use the NG preferences sheet. |  |
| The staff will remove the cap from the end of the NG tube. |  |
| The staff will attach a syringe at the end of the NG tube. The staff will pull on the syringe to collect liquid from my stomach. |  |
| Sometimes the NG tube is not in the right place, the staff will adjust it so that it reaches my stomach. |  |
| The staff will check that I am feeling comfortable. |  |
| Then the staff will attach a syringe to the end of the NG tube. |  |
| The staff will push water through to flush the tube. |    |
| The staff will then push the liquid food through the NG tube into my stomach. Usually, it will take around 5 minutes. |  |
| The staff will follow the preferences for supporting me during my NG feed. |  |
| Once the liquid food has been given, the staff will flush the NG tube again with water. |    |
| The staff will then disconnect the syringe and put the cap back on the end of the tube. |  |
| The NG feed is finished. |  |
| I can now return to the communal area. |  |

# Support during Naso-Gastric feeding

NG feeding can be overwhelming. We want you feel as safe and supported. You can go through this form with staff to work out how best to support you **before, during** and **after an NG feed**. You can **come back to this form** and change your answers at any time. If you're not sure what might help, that’s okay — we can work this out together.

The list below is not the only ways you can be supported. If you have an idea that you don’t see here, speak to us and we can see what we can do.

We will always do **our best to listen** and **respond to your preferences** **where it’s** **safe and possible.**

# Preferences before, during and after NG feed

### Before the NG feed,

- ☐ I want to my NG tube to already be fitted before the feed.
- ☐ I want to have my NG to be fitted as part of the NG feed procedure.
- ☐I want to be given medication beforehand.
- ☐I want to have a 5-minute warning before the NG feed
- ☐ Other (please specify):

## Going to the NG feed

- ☐I want to go from the dining room through to the treatment room for the feed
- ☐I want to go from the communal space to the treatment room for the feed.
- ☐I will walk in by myself
- ☐I will walk in with support from a member of staff
  - ☐ Hold my hand
  - ☐ Guide me to the treatment room
  - ☐ Not to speak to me
  - ☐ To remind me of why
  - ☐ Hold my hand
- ☐ Other (please specify):

_______________________________________________________

### During the NG feed:

- ☐ Lighting preferences in the treatment room (please specify)

_______________________________________________________

- ☐ Staff to talk me through what’s happening
- ☐ Staff to stay quiet unless needed
- ☐Staff to distract me with talking
- ☐ A specific staff member to talk (please name if you'd like):

_______________________________________________________

- ☐ Visual prompts (e.g., grounding images, calming photos)
- ☐ Breathing exercises (guided or self-directed)
- ☐Distractions
  - ☐ Counting
  - ☐ Tapping
  - ☐ Watching a video
  - ☐ Music
  - ☐ Radio
- ☐ Gentle music, calming sounds or the radio (please specify)

_______________________________________________________

- ☐ Silence.
- ☐ A fidget or grounding object (if safe to use)
- ☐ Other (please specify):

_______________________________________________________

- Timing prompt to let me know how long is left
- ☐ Countdown
- ☐ Timer
- ☐ Verbal prompts (e.g., Almost there, About halfway)

After the NG feed:

- ☐ I want to be left alone
- ☐ I want a staff to reassure me
- ☐ I want to play a game
- ☐ I want to watch my favourite TV show
- ☐ To have a visit or phone call
- ☐ Gentle music or calming sounds
- ☐ A fidget or grounding object (if safe to use)
- ☐ Other (please specify):

Other preferences we should consider about the NG feed (please specify):

______________________________________________________

Naso-Gastric support plan

Young person:

Date:

These are my preferences for support before, during and after an NG feed. Please accommodate these preferences as far as clinically possible.

| Before | |
| --- | --- |
| Do: | Don’t |
|  |  |
| During | |
| Do: | Don’t |
|  |  |
| After | |
| Do: | Don’t |
|  |  |

# Strategies that can help me throughout the process

Breathing Exercise

Focusing on our breathing can be a great tool to lower levels of anxiety and calm our nervous system. Here is an exercise you can try:

Ensure that you are sitting on a comfortable chair or laying on a bed.

Take a breath in for 4 seconds (through the nose if possible)

Hold the breath for 2 seconds

Release the breath taking 6 seconds (through the nose if possible) then pause slightly before breathing in again.

Grounding Exercise

The **5,4,3,2,1** method helps bring our awareness back to the present moment when we’re feeling anxious.

- What are **5** things you can see?
- What are **4** things you can touch?
- Can you notice **3** things you can hear?
- Can you recognise **2** things you can smell?
- What is **1** affirmation that could help how you’re feeling right now?

Grounding object

Having a grounding object that you can hold and focus on when you feel anxious can be helpful. For example, a fidget toy or a piece of fabric.

Imagery

When our minds are active, it can be helpful to engage in mindfulness to help calm anxiety. Imagining ourselves in a peaceful setting can be a distraction from things causing us stress.

Reasons to Recover

It can be helpful to keep a list of your reasons to recover and remind yourself of this when things feel difficult. These can be future goals, things you would like to get back to or hobbies.
